# Applicability of the d-Band Model to Predict the Influence of Elastic Strains on the Adsorption Energy of Different Adsorbates onto Pt and PtO_2_ Surfaces

**DOI:** 10.1021/acsomega.4c03830

**Published:** 2024-06-24

**Authors:** Carmen Martínez-Alonso, Javier LLorca

**Affiliations:** † IMDEA Materials Institute, C/Eric Kandel 2, Getafe, 28906 Madrid, Spain; ‡ Department of Inorganic Chemistry, Complutense University of Madrid, 28040 Madrid, Spain; § Department of Materials Science, Polytechnic University of Madrid, E. T. S. de Ingenieros de Caminos, 28040 Madrid, Spain

## Abstract

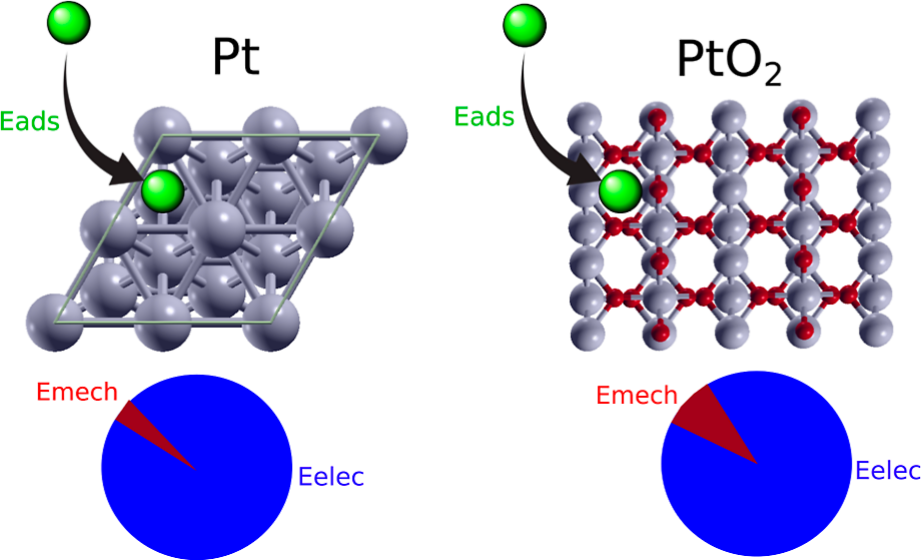

The influence of elastic strains on the adsorption processes of seven adsorbates (H, C, N, O, CO, NO, and H) onto the surface of Pt(111) and PtO_2_ (110) has been investigated using density functional theory (DFT) simulations. The total adsorption energy was decomposed into mechanical and electronic contributions. Our results indicate that elastic strain in metals affects the adsorption energy by modifying the electronic structure of the surface rather than changing the physical space where the atoms reside after adsorption. In fact, the mechanical contribution to the adsorption energy in Pt was negligible compared to the electronic interaction and independent of the deformation. The mechanical contribution in the case of PtO_2_ was also independent of the applied strain, but its magnitude was slightly higher due to the ionic bonding between Pt and O atoms in the slab. The variation of the electronic contribution to the adsorption energy in Pt and PtO_2_ followed the predictions of the d-band model for all adsorbates, expanding its applicability to different adsorbates onto the same surface and to oxides.

## 1 Introduction

The development of new catalysts constitutes a considerable challenge, and thousands of scientists around the world are dedicated to this task.^1,2^ In this regard, the electronic structure calculations in general, and density functional theory (DFT) simulations in particular, play a very important role in many aspects of this endeavor:^3^ helping to determine the mechanism of the different reactions that occur during the catalysis process,^4^ understanding how modifications in the reaction conditions affect the products,^5−7^ and overall achieving a complete description of the reaction pathway.^8,9^ These computational methods, together with the computing power afforded by modern supercomputing facilities, have been successfully employed for the high-throughput search of new catalysts.^10−12^

Traditionally, the search for new heterogeneous catalysts has been carried out in two principal axes: the addition of alloying elements and the introduction of defects or changes in the surface orientation. Alloying can improve the electrocatalytic performance by chemically modifying the electronic structure of the material.^13,14^ In contrast, the presence of defects or alterations in the surface orientation can change the intrinsic electronic properties depending on the material. In some cases, these mechanical changes allow molecules to approach the surfaces at different angles, facilitating the reaction^15,16^ without changing the electronic structure, while in specific MOFs or oxides, introducing vacancies can significantly change the electronic properties.^17,18^ In recent years, a new axis for the development of catalysts has been explored: elastic strain engineering. It has been shown both experimentally^19^ and computationally^20^ that the application of large mechanical strains over material surfaces can modify the catalytic properties of materials, even breaking the ever-present scaling relations in catalysis.^21^ Moreover, DFT calculations reveal that the mechanism behind the change in said catalytic properties is the alteration of the electronic structure of the material, as evidenced in the displacement of the corresponding d-bands.^22^ In other words, elastic strain engineering enables the modification of the chemical characteristics of metallic surfaces by the use of mechanical forces.

In this regard, the d-band center model proposed by Hammer and Norskov^23,24^ explains the modification of the electronic surfaces and the consequent variations in the adsorption energy for different metals. According to this theory, the d-band center shift produced by the elastic strains can be used to quantify the variation of adsorption energy in different surfaces.^22^ It shifts up or down when applying tensile or compressive strains, respectively, in order to maintain a constant filling. As a general rule, the application of tensile (compressive) strains in metals with more than half-filled d-bands reduces (increases) the d-orbital overlap due to a sharpening of the d-band producing a strengthening of the adsorption process (more negative adsorption energies). The opposite phenomenon occurs for metals with less than half-filled d-bands.^25^ The d-band model has been verified for different adsorption surfaces for a given adsorbate^23,24,26^ but the applicability of this theory to analyze the influence of elastic strains on the adsorption energy of different adsorbates on the same surface has not been analyzed in detail.

In this investigation, the effect of elastic strains on the adsorption energy of seven different adsorbates (H, C, N, O, CO, NO, and OH) on Pt and PtO_2_ is studied. The aim is to compare a pure metal—Pt—with an oxide containing the same transition metal—PtO_2_—to analyze the influence of oxygen atoms on the surface in the mechanical and electronic contributions to the adsorption energy. So far, the effect of elastic strains on platinum has been considered,^19,20,22,27^ but the application of strain on transition metal oxides is unexplored in the adsorption of H, C, N, O, CO, NO, and OH. The applicability of the pd-band theory to rutile-type structures has not been quantified. The electronic and mechanical contributions to the adsorption process are decoupled, following the strategy proposed by Francis and Curtin,^28^ to provide a better understanding of the processes. It is found that the mechanical contribution to the adsorption process is independent of the applied elastic strain, and the energy associated with this contribution is marginal in most cases, particularly in the case of Pt. The elastic strains do influence, however, the electronic contribution, and the energy associated with the mechanism decreases with tensile strains and increases with compressive strains for all adsorbates in Pt and PtO_2_. These trends and the magnitude of the energy change agree with the d-band model. This study expands the applicability of the d-band theory model to different adsorbates on metallic and oxide surfaces and provides a deeper analysis of the adsorption processes with and without the application of elastic strains.

## 2 Methodology

### 2.1 Adsorption Energies

The adsorption process of seven different adsorbates (H, O, OH, C, CO, N, and NO) on a Pt(111) surface and on a PtO_2_(110) surface under the application of elastic strains is studied by means of DFT simulations. The adsorption surfaces were chosen for being the lowest in energy for Pt and PtO_2_. Atomic simulation environment (ASE)^29^ was used to generate (2 × 2) slab supercells with four layers of atoms perpendicular to the surface from the equilibrium lattice parameters previously calculated. The slab for PtO_2_ is constructed from an orthorhombic bulk geometry. Slabs were separated by 10 Å of vacuum perpendicular to the surface and submitted to periodic boundary conditions. The coverage of all the chemical species was 1/4 monolayers. The top two layers of the metal and oxide surfaces and all adsorbed atoms were fully relaxed, whereas the two bottom layers were fixed to simulate the bulk.

The adsorption energy of each different adsorbate *X* was defined as
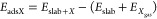
1where *E*_slab_ and *E*_slab+*X*_ stand for the total energies of the slab without and with the adsorbate *X*. *E*_*X*_gas__ accounts for the total energy of the molecules in the gaseous state. The specific equations for each of the seven adsorbates and the corresponding adsorption energies can be found in Sections S1–S5 of the Supporting Information.

### 2.2 Mechanical and Electronic Decoupling

**Figure 1:**
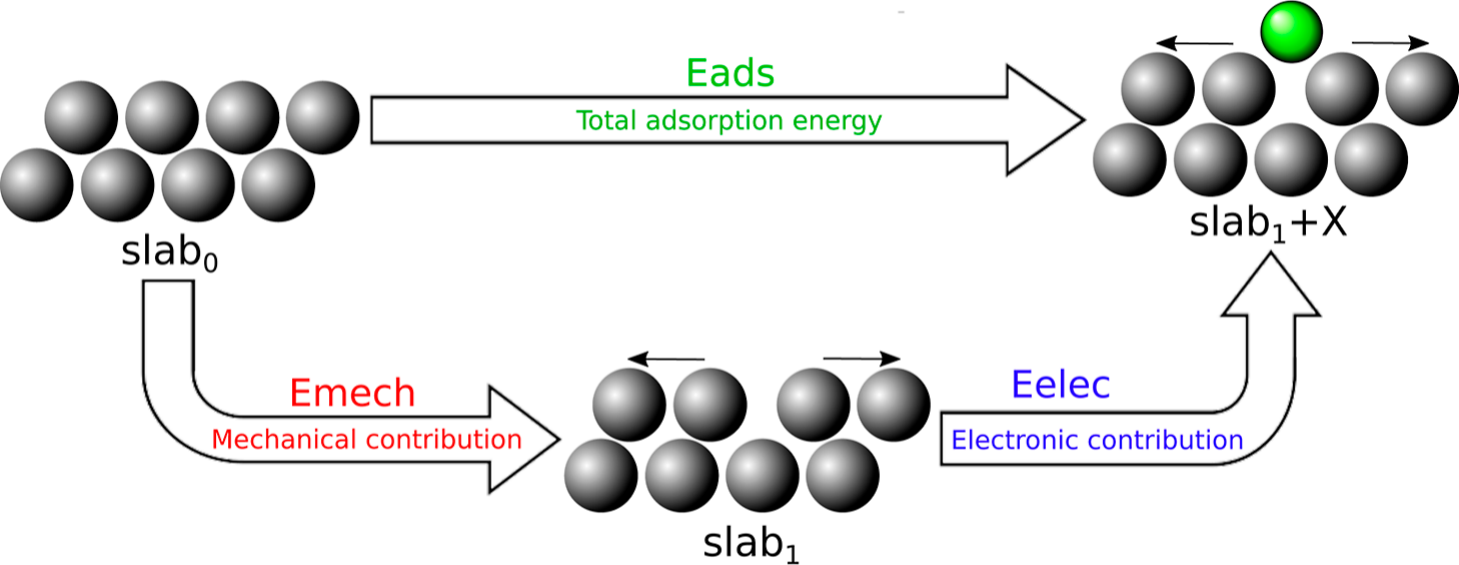
Illustration of the separation in electronic and mechanical contributions for the adsorption process. slab_0_ is the initial surface undeformed, and slab_1_ is the final surface deformed by the adsorbate. The black arrows represent the atomic displacements of the substrate atoms of the first layer of the supercell.

In order to achieve a deeper understanding of the effect of elastic strains in the adsorption process, we separated the adsorption energy into two contributions, electronic and mechanical, following refs (21 and 28). The electronic contribution, *E*_elec_, accounts for the interactions between the adsorbate atoms and the substrate surface atoms at the adsorption distance, while the mechanical contribution, *E*_mech_, includes the elastic energy associated with the surface deformations due to the adsorbate’s presence. Following Figure 1, the sum of both contributions gives the total adsorption energy defined in eq 1
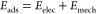
2where *E*_elec_ stands for the difference in energy due to the presence of the adsorbate once the surface of the slab has been relaxed, whereas *E*_mech_ is calculated from the difference between the deformed and undeformed slab without the adsorbed atom. They are given by
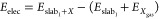
3
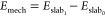
4where  is the energy of the initial surface undeformed and  is the energy of the final surface deformed by the adsorbate.

### 2.3 Application of Strains

The surface slabs were subjected to biaxial stresses in parallel to the surface plane. The deformation gradient **F** applied to the supercell was
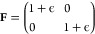
5where ϵ stands for the normal strain along *x* and *y* directions. The stress perpendicular to the slab surface was zero.

The limits of mechanical stability for surface supercells under strain were investigated by analyzing the harmonic phonon spectra with the Phonopy code.^30^ Taking into account the theoretical values of the stability limits obtained with the phonon calculations in our previous studies,^20,22^ it was decided to explore the effect of elastic strains on the adsorption energies in a range of −5% biaxial compression to 5% biaxial tension to guarantee the stability of the surfaces. The harmonic phonon spectra can be found in the Supporting Information in Figure S1. Uniaxial strains were not considered in this study, as we evidenced in our previous paper^22^ that they induce approximately half of the effect of biaxial strains in the adsorption energy and that the tendencies are proportional.

The equations of the two contributions to the adsorption energy (electronic and mechanical) after the application of strain are given by
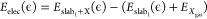
6
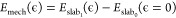
7

### 2.4 Computational Details

The DFT plane wave simulations were performed using the GPU-enabled version of the Quantum Espresso package.^31−33^ The calculations were carried out using ultrasoft pseudopotentials, while the electronic exchange–correlation was described using the generalized gradient approximation (GGA) with the Perdew–Burke–Ernzerhof (PBE) functional.^34^ In order to test if the PBE functional was accurate enough to calculate the adsorption energy, the metaGGA SCAN^35^ was also considered, as it is known to provide accurate values of the adsorption energies at a significantly higher computational cost. The differences in *E*_adsH_ between the generalized gradient approximation (GGA) with the Perdew–Burke–Ernzerhof exchange–correlation functional and the metaGGA SCAN functional were only 0.03 eV. In addition, the effect of biaxial elastic strains in the adsorption energy of H, O, and OH on a Pt(111) slab is plotted in Figure S2 for GGA-PBE and metaGGA-SCAN functionals. Although there are some differences in adsorption energy between both functionals, the trends with strain are identical. Additionally, GGA functionals have been proven accurate for metal and metal-oxide surfaces in the literature.^36,37^ That moves us to conclude that utilizing a GGA functional is sufficient for the aim of this work. A cutoff energy of 80 Ry was selected for the plane-wave basis, and the Monkhorst–Pack *k*-points were sized (20 × 20 × 20) for the bulk calculations and (4 × 4 × 1) for the slabs supercells. All calculations include spin-polarization corrections. Brillouin zone calculations were performed using a Marzari–Vanderbilt–De Vita–Payne cold smearing of 0.015 Ry.^38^ After a proper convergence study, all these parameters have been selected and matched the parameters used in similar studies.^39−41^

**Figure 2:**
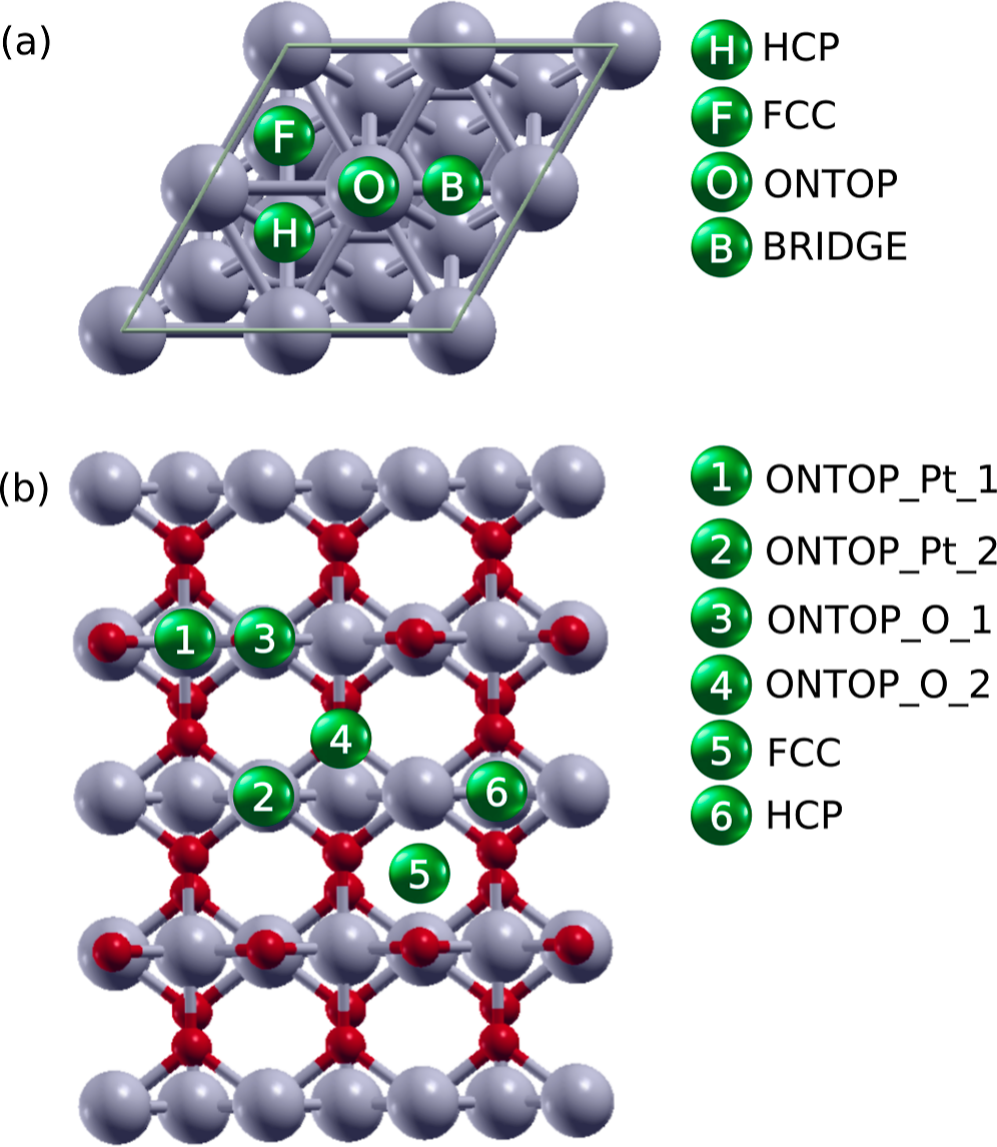
Different adsorption positions in (a) Pt(111) and (b) PtO_2_(110).

DFT calculations of adsorption were carried out in the four possible adsorption sites for Pt(111) (Figure 2a), whereas adsorption can take place in six different positions for PtO_2_(110) surface (Figure 2b).

Finally, the charge donated from/to the surface to/from the seven different adsorbates in Pt and PtO_2_ was computed. The Bader program, developed by Yu and Trinkle^42^ was used to this end. This code uses the Bader definition of an atom, a 3-D object delimited by surfaces having zero flux in the electron density gradient vector field.^43^ More specifically, we analyzed the electron density of each of the atoms on the surface and the adsorbate, and the differences with respect to the standard charge revealed the electronic transfers.

## 3 Results

### 3.1 Pt

Seven molecules (H, O, OH, C, CO, N, and NO) were adsorbed onto Pt fcc(111) to understand the effect of the adsorbates in the electronic-mechanical decoupling under strain. The most favorable adsorption site was the FCC position in all cases (Figure 2a). The diatomic adsorbates OH, CO, and NO were adsorbed perpendicularly to the metallic slab with O, C, and N atoms closer to the surface, respectively.

The corresponding adsorption energies at the FCC sites and the electronic and mechanical contributions are detailed in Table 1. The adsorption energies of OH, N, and C are positive, while those of H, O, NO, and CO are negative. The range of energy values associated with the adsorption process varies from 2.01 eV for carbon to −2.16 eV for oxygen. The mechanical contribution is always positive because it is associated with the elastic accommodation of the atoms in the surface of the slab around the adsorption site (Figure 1). Our results show that this contribution is much smaller (in the range of 0.04–0.21 eV) than the electronic one that dominates the total adsorption energy for most adsorbates. The only exception is N, where both *E*_elec_ and *E*_mech_ are small, similar, and positive. The mechanical energy (*E*_mech_) increases with the atomic radius (H < O ∼ N < C) for monatomic adsorbates. However, the trend is not followed for diatomic adsorbates (CO < NO < OH). Here, *E*_mech_ is inversely proportional to the bond length (O–H: 90 pm, N–O:115 pm, C–O: 140 pm).

**Table 1:** Total Adsorption, Electronic, and Mechanical Energies for the Adsorption of H, O, OH, N, NO, C, and CO in FCC Sites of Pt fcc(111) at Zero Strain

system	*E*_ads_ (eV)	*E*_elec_ (eV)	*E*_mech_ (eV)
H/Pt	–0.49	–0.53	0.04
O/Pt	–2.16	–2.31	0.15
OH/Pt	1.19	1.00	0.19
N/Pt	0.27	0.13	0.14
NO/Pt	–2.04	–2.17	0.12
C/Pt	2.01	1.80	0.21
CO/Pt	–1.74	–1.82	0.08

**Figure 3:**
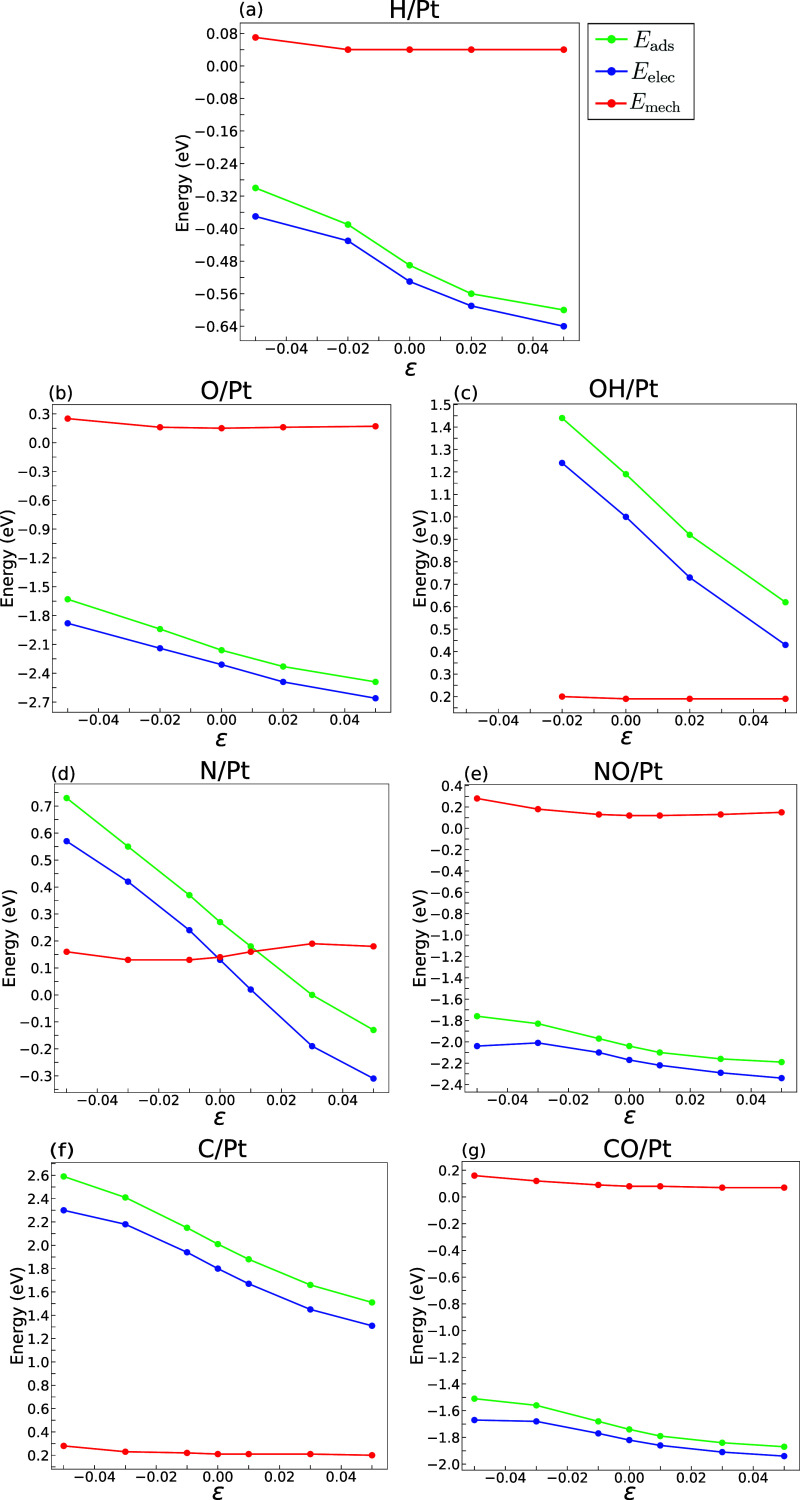
Different contributions for the adsorption of (a) H, (b) O, (c) OH, (d) N, (e) NO, (f) C, and (g) CO into a Pt fcc(111) surface. The green, blue, and red lines represent the total adsorption energy (*E*_ads_), as well as the electronic (*E*_elec_) and mechanical (*E*_mech_) contributions, respectively. Data at −5% compression in (c) was omitted due to the instability of the slab.

The total adsorption energy, as well as the electronic and mechanical contributions, for the seven adsorbates onto mechanically strained Pt, from −5% compression to 5% tension, is plotted in Figure 3. Here, in agreement with our previously reported results,^22^*E*_ads_ (represented in green) depends on the applied biaxial elastic strain: decreases with tensile strains and increases with compressive ones. However, it is worth noting that the effect of elastic strains on the adsorption energy differs greatly among adsorbates. The adsorption energy of CO (Figure 3g) or NO (Figure 3e) only shifts by about 0.2 eV between −5 and 5% biaxial strain, but the variation of the adsorption energy in C (Figure 3f) and N (Figure 3e) is more than three times larger in the same strain range.

The information in Figure 3f also includes the variation of the electronic and mechanical contributions to the adsorption energy under the application of external elastic strains. Remarkably, *E*_mech_ (red lines) remains practically constant with strain in all cases, while the sensitivity of the adsorption energy to the applied elastic strains is dictated by *E*_elec_ (blue lines). This result indicates that the changes in the adsorption energy with the application of strain are primarily due to the electronic adsorbate–substrate interaction and that the mechanical accommodation of the adsorbate on the surface plays a minor role from the energy viewpoint.

**Figure 4:**
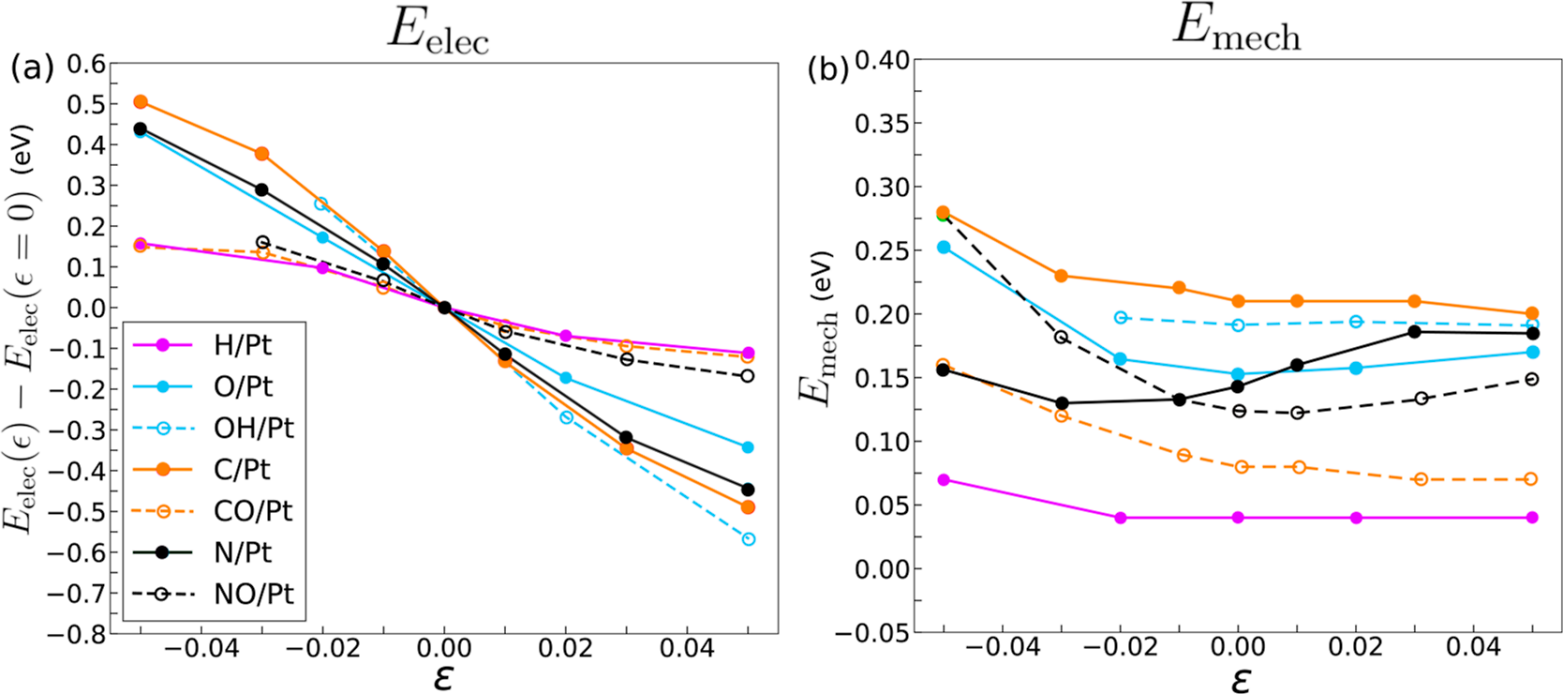
(a) Electronic and (b) mechanical contributions to the adsorption energies for different adsorbates onto Pt fcc(111) as a function of the applied strain. The electronic contribution values are referenced with respect to the ones corresponding to zero strain. Solid markers and lines represent monatomic adsorbates, while dash lines and empty circles illustrate diatomic molecules.

The variation of the electronic and mechanical contributions to the adsorption energy is plotted as a function of the applied biaxial elastic strain in Figure 4. The electronic contribution is referenced with respect to the situation where no strain is applied [*E*_elec_(ϵ) – *E*_elec_(ϵ = 0)] in order to facilitate its visualization. The information in Figure 4a shows that the influence of mechanical strains on the electronic contribution to the adsorption energy (and, thus, on the total adsorption energy) follows the order C > OH > N > O > NO > CO > H. In particular, it is maximum for O, C, N, and OH and minimum for H, NO, and CO. This illustrates how the presence of an adsorbate with a higher electronic effect produces bigger displacements in the atoms of the surface, reflected in an increment of *E*_mech_.

### 3.2 PtO_2_

The role of electronic and mechanical contributions to the adsorption energy was also analyzed in the adsorption process of H, O, OH, C, CO, N, and NO onto a PtO_2_(110) surface. It was found that the most favorable adsorption site for H and N was on top of an oxygen atom (position 3: ONTOP_O_1 in Figure 2b). In contrast, C, CO, NO, O, and OH get adsorbed on top of a Pt atom (position 2: ONTOP_Pt_2 in Figure 2b). The values for *E*_ads_, *E*_elec_, and *E*_mech_ are shown in Table 2.

**Table 2:** Total Adsorption, Electronic, and Mechanical Energies for the Adsorption of H, O, OH, N, NO, C, and CO in the Optimum Adsorption Site of PtO_2_(110) at Zero Strain

system	*E*_ads_ (eV)	*E*_elec_ (eV)	*E*_mech_ (eV)
H/PtO_2_	–1.01	–1.05	0.04
O/PtO_2_	–0.42	–0.88	0.46
OH/PtO_2_	1.74	1.24	0.50
N/PtO_2_	2.53	2.07	0.46
NO/PtO_2_	–0.70	–1.30	0.60
C/PtO_2_	4.88	4.30	0.58
CO/PtO_2_	–1.42	–1.94	0.53

**Figure 5:**
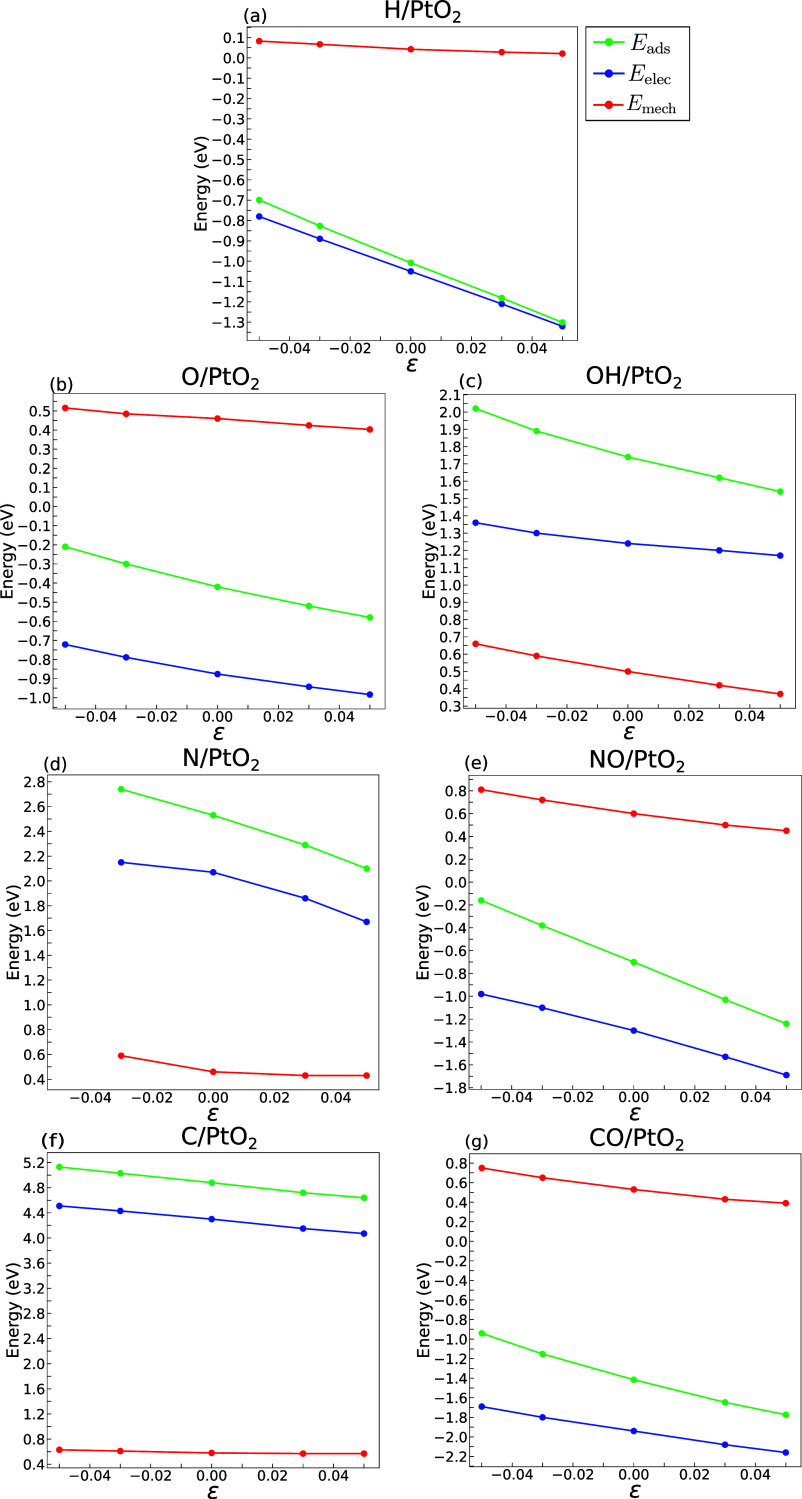
Different contributions for the adsorption of (a) H, (b) O, (c) OH, (d) N, (e) NO, (f) C, and (g) CO into a PtO_2_ (110) surface. The green, blue, and red lines represent the total adsorption energy (*E*_ads_), as well as the electronic (*E*_elec_) and mechanical (*E*_mech_) contributions, respectively. Data at −5% compression in (d) was omitted due to the instability of the slab.

The total adsorption energy (green), the electronic contribution (blue), and the mechanical contribution (red) of the 7 adsorbates in a PtO_2_(110) surface are plotted in Figure 5. As in the case of Pt(111), the total adsorption energy decreased with the application of biaxial tensile strains and increased with compressive ones. It should be noted, however, that the mechanical contribution to the total adsorption energy is in the range of 0.5–0.7 eV for most adsorbates (the only exception is H) in the case of PtO_2_(110) surfaces. These values are much higher than those found in Pt(111) surfaces.

**Figure 6:**
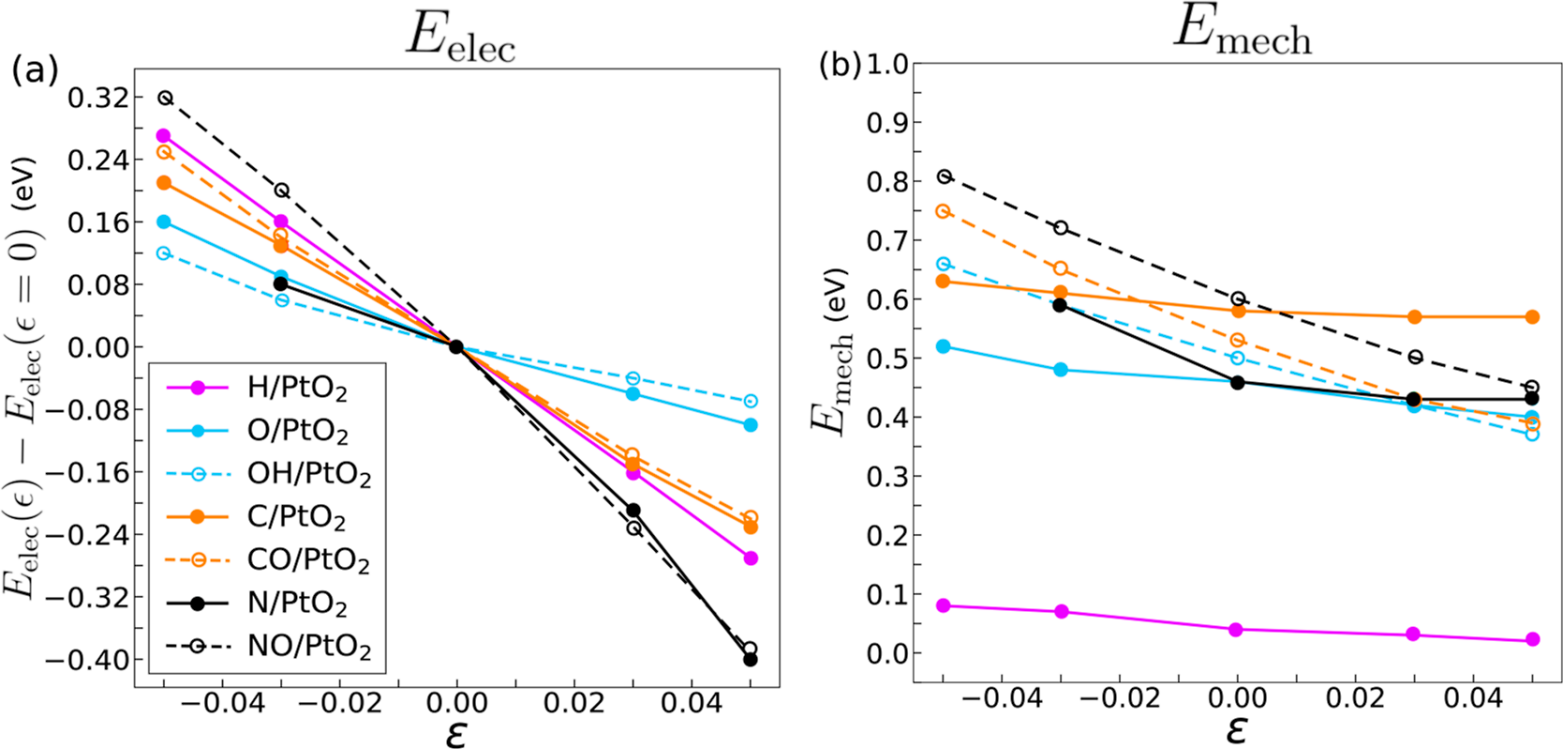
(a) Electronic and (b) mechanical contributions to the adsorption energies for different adsorbates onto PtO_2_(110) as a function of the applied strain. The electronic contribution values are referenced with respect to the ones corresponding to zero strain. Solid markers and lines represent monatomic adsorbates, while dash lines and empty circles illustrate diatomic molecules.

The differences in the electronic and mechanical contributions to the adsorption energy in PtO_2_(110) with strain are plotted in Figure 6 for the 7 different adsorbates. The electronic contribution in Figure 6a is referenced with respect to the situation where no strain is applied [*E*_elec_(ϵ) – *E*_elec_(ϵ = 0)]. The variation of *E*_elec_ is almost linear with the applied biaxial elastic strain: compressive strains increase the adsorption energy, which decreases with tensile strains. The slope of the quasi-linear relationship between *E*_elec_ and ϵ follows the order NO > N > H > CO > C > O > OH different from the one found on Pt(111). The maximum sensitivity to the elastic strains is found in N and NO, while the minimum is reported for O and OH. Overall, the comparison of Figures 4a and 6a indicates that the electronic contribution to the adsorption energy is more sensitive to the applied elastic strain in the case of Pt(111), as compared with PtO_2_.

Regarding the mechanical contributions to the adsorption energy (Figure 6b), they are constant (or decrease slightly with the tensile strains) in all adsorbates and higher than those found in Pt(111) (Figure 4b) in all cases except for H, in which the mechanical contribution to the adsorption energy is around 0.5 on Pt(111) and PtO_2_(110).

## 4 Discussion

The results presented above show large differences in the variation of *E*_elec_ (which provides the main contribution to the adsorption energy) with the applied strain for different adsorbates in both Pt and PtO_2_. This behavior can be rationalized through the d-band model and the surface charge transfer from/to the adsorbate. As previously mentioned, the d-band center acts as an excellent estimator of the effect of elastic strains on metallic surfaces.^22^ However, its role in the description for different adsorbates onto the same system has not been quantified. Complementary, the extension of the d-band theory to the so-called pd-band model in oxides is scarcely explored. The charge transfer from/to the surface to/from the adsorbate is a common approach to elucidate the nature of the interaction as well as the effect of strain.^44−47^

### 4.1 Adsorbate Effect in the Variation of *E*_elec_ with Strain on Pt

The effect of elastic strains on the adsorption energy of different transition metals has been rationalized through the d-band model.^23^ As it is widely known, the changes in the electronic structure can be quantified by the projected density of the states (PDOS).^24^ Hammer and Norskov proposed the theory of the d-band center to explain the adsorption of hydrogen atoms on the surface of nickel, copper, platinum, and gold.^23^ It rationalizes the adsorption energy of the process as a function of the d-band center shift. However, the applicability of this model to explain the influence of different adsorbates on the strain elastic sensitivity has not been analyzed. To this end, the projected density of the states (PDOS) of the d-band onto the Pt surface atoms was analyzed for all adsorbates, and the d-band center shift was calculated.

On the pristine Pt(111) and PtO_2_(110) surfaces, changes in the d-band/p-band centers occur in accordance with the d-band center theory.^23^ This theory suggests that the overlap between d and p-states at adjacent sites will either expand or contract when a surface experiences compressive or tensile strains. Consequently, the d and p-bandwidths adjust to ensure a consistent filling level. Hence, compressive or tensile strains induce downward or upward shifts in the d and p-band centers, respectively, as stated in the literature^25,48^ and demonstrated in our prior publication.^22^ The shifts in the Fermi energy(*E*_f0_ – *E*_f_) and the d-band center (*E*_d0_ – *E*_d_) are shown in Table 3 for all the adsorbates, while the full PDOS for the different adsorbates can be found in Figure S3 of the Supporting Information. In all cases, the Fermi energy shifts toward higher energies, whereas the d-band center shifts down. This phenomenon reveals a higher occupancy in the bonding states due to the adsorbate–surface interaction. The d-band center shift allows to rationalize the variations of *E*_elec_ with the applied elastic strain. The adsorbates that lead to a maximum shift in the d-band center, expressed by *E*_d0_ – *E*_d_, also show the largest variation in *E*_elec_ with the elastic strain (i.e., C, N, O, and OH). On the contrary, if the change in the d-band center is minimal in the presence of adsorbate (i.e., H, CO, and NO), it also shows the lowest influence of the elastic strains in *E*_elec_ (Figure 4a). Moreover, the Fermi energy (*E*_f_) and the d-band center (*E*_d_) are illustrated in Table 4 for the seven X/Pt(111) systems subjected to −3% biaxial compression and 5% biaxial tension. In accordance with the d-band model,^23,24,49^ tensile strains narrow the d-band and lead to an upshift of *E*_d_ to maintain the d-filling constant, while compressive strains make the bands wider and move the d-band center down. The behavior of all adsorbates follows this behavior.

**Table 3:** Electronic Shifts in Pt(111) Due to the Adsorption of the 7 Adsorbates at Zero Strain^a^

system	*E*_f_ (eV)	*E*_f0_ – *E*_f_ (eV)	*E*_d_ (eV)	*E*_d0_ – *E*_d_ (eV)
Pt(111)	3.57	0.00	–2.09	0.00
H/Pt(111)	3.66	–0.09	–2.22	0.13
O/Pt(111)	3.70	–0.14	–2.52	0.43
C/Pt(111)	3.75	–0.18	–2.59	0.5
N/Pt(111)	3.70	–0.13	–2.55	0.46
OH/Pt(111)	4.30	–0.73	–2.45	0.36
CO/Pt(111)	3.61	–0.04	–2.23	0.14
NO/Pt(111)	3.77	–0.20	–2.38	0.29

a *E*_f_, *E*_f0_ – *E*_f_, *E*_d_, and *E*_d0_ – *E*_d_ stand for the Fermi energy, the shift in the Fermi energy with the adsorption of the corresponding adsorbate, the d-band center, and the shift in the d-band center after the adsorption, respectively. All values are expressed in eV.

**Table 4:** Fermi Energy (*E*_f_) and d-Band Center (*E*_d_) for the X/Pt(111) Systems under Biaxial Strain

system	strain (%)	*E*_f_ (eV)	*E*_d_ (eV)
H/Pt(111)	–3	3.87	–2.31
	0	3.66	–2.22
	5	2.79	–2.00
O/Pt(111)	–3	3.91	–2.56
	0	3.70	–2.52
	5	2.85	–2.31
C/Pt(111)	–3	3.94	–2.65
	0	3.75	–2.59
	5	2.91	–2.49
N/Pt(111)	–3	3.87	–2.65
	0	3.70	–2.55
	5	2.92	–2.49
OH/Pt(111)	–3	4.53	–2.46
	0	4.30	–2.45
	5	3.37	–2.10
CO/Pt(111)	–3	3.80	–2.32
	0	3.61	–2.23
	5	2.76	–2.06
NO/Pt(111)	–3	3.97	–2.39
	0	3.77	–2.38
	5	2.70	–2.16

In addition to the shift of the d-band, the charge transfer between an adsorbate and a surface can shed light on the nature of the interaction. The charge transfers for the different adsorbates in Pt fcc(111) are depicted in Table 5. A negative charge transfer stands for a loss of electronic density, whereas a positive charge transfer represents a gain of electronic density. The four platinum atoms of the model surface are numbered and Pt1, Pt2, and Pt3 are the atoms next to the adsorption site. The charge donated from the surface to the adsorbate is given as a single value for monatomic adsorbates, while the charge of each atom and the total charge of the adsorbate are shown for the molecular adsorbates.

**Table 5:**
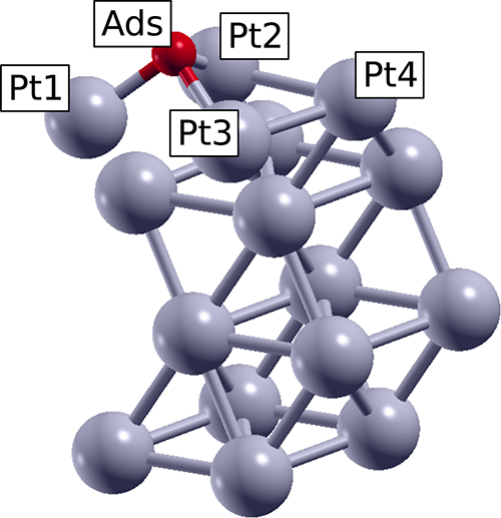
Charge Transfer for the 7 Adsorbates in a Pt fcc(111) Surface at FCC Position at Zero Strain^a^

system	Pt1 (*e*)	Pt2 (*e*)	Pt3 (*e*)	Pt4 (*e*)	adsorbate (*e*)	
H/Pt(111)	–0.02	0.04	0.02	0.00	0.11	
N/Pt(111)	–0.26	–0.30	–0.28	0.06	0.80	
O/Pt(111)	–0.25	–0.28	–0.26	0.06	0.80	
C/Pt(111)	–0.17	–0.21	–0.19	0.06	0.50	
OH/Pt(111)	–0.14	–0.16	–0.16	0.08	1.09 (O)	0.47
					–0.62 (H)	
CO/Pt(111)	–0.07	–0.11	–0.08	0.05	–1.69 (C)	0.29
					1.98 (O)	
NO/Pt(111)	–0.16	–0.20	–0.17	0.07	0.21 (N)	0.50
					0.29 (O)	

a The Pt1, Pt2, Pt3, and Pt4 positions are shown in the scheme at the side of the table. All values are expressed in elemental units where *e* = −1. A positive value expresses a gain of negative charge, whereas a negative value expresses a loss of negative charge.

In all cases, the Pt surface donates electronic density to the adsorbate. The H atom receives the least charge, while O and N receive the highest one, in agreement with the different electronegativities of each element. For the molecular adsorbates, the electronic density gained from the surface is distributed among both atoms in the case of OH, CO, and NO, following their corresponding electronegativity, and O atoms receive the majority of the charge.

If the same atoms of the monatomic and diatomic adsorbates are compared, it is found that variation of *E*_elec_ with the applied strain increases with the amount of charge donated from the surface to the atom of the adsorbate closest to the surface. That is, the O adsorbate gets 0.8*e* from the surface, while the O atom of the OH adsorbate gets 1.09*e* from both the surface and the H atom, and the variations of *E*_elec_ with strain for OH are higher than for O. Similar conclusions can be reached for C and CO as well as N and NO, as shown in Figure 4a. This corroborates the conclusions obtained from the PDOS.

The analysis of the effect of elastic strains on the charge transfer led to a familiar result: tensile strains increase the distance of the surface atoms and make the area of the adsorption hole bigger. As a consequence, the distance between the surface and the adsorbate decreases, and the charge transfer increases. Conversely, compressive strains increase the adsorbate–surface distance and minimize the charge transfer. The variations in charge with strain for the seven adsorbates in Pt(111) are shown in the Table S5. Albeit important, the variation of charge transfer as a function of mechanical strain (from −3% compression to 5% tension) is small and similar for all adsorbates.

### 4.2 Adsorbate Effect in the Variation of *E*_elec_ with Strain on PtO_2_

Following the same methodology as for Pt, the band center shifts when the adsorption occurred on a surface of PtO_2_(110) were analyzed for the 7 adsorbates. In this case, both the d-bands of the Pt atoms and the p-bands of the O atoms (*E*_pd_) on the surface were analyzed.

The Fermi energy (*E*_f_), the band center (*E*_pd_), and their shifts with respect to the clean slab for the different adsorbates are depicted in Table 6. In addition, the full PDOS for the different adsorbates can be found in Figure S4 of the Supporting Information. All the Fermi energies shift toward higher energies, while the band centers shift down in the presence of adsorbates, as was the case for the adsorption on Pt(111). This behavior is in agreement with the phenomenon of adsorption and reflects the higher occupancy in the bonding states due to the presence of the adsorbates on top of the surface. Again, the shift of the band center explains the variation of *E*_elec_ with the elastic strain. Large magnitudes of *E*_pd0_ – *E*_pd_ lead to a more marked effect of the elastic strains on *E*_elec_ (Figure 6a). In particular, the variation in *E*_elec_ with the elastic strains followed the order NO > N > H > CO > C > O > OH, while the band shift magnitude was N > NO > H > CO > C > OH > O. Thus, the only exceptions to this rule were N/NO and O/OH, whose order was exchanged, but it should be noted that the differences in the calculated adsorption energies were very small and within the error of the DFT simulations.

**Table 6:** Electronic Shifts in PtO_2_(110) with the 7 Adsorbates at Zero Strain^a^

system	*E*_f_	*E*_f0_ – *E*_f_	*E*_pd_	*E*_pd0_ – *E*_pd_
PtO_2_(110)	2.21	0.00	–1.70	0
H/PtO_2_(110)	3.06	–0.85	–2.17	0.47
O/PtO_2_(110)	2.22	–0.01	–1.87	0.17
C/PtO_2_(110)	2.57	–0.36	–2.06	0.36
N/PtO_2_(110)	2.96	–0.75	–2.37	0.67
OH/PtO_2_(110)	2.66	–0.45	–1.94	0.24
CO/PtO_2_(110)	2.86	–0.65	–2.13	0.43
NO/PtO_2_(110)	3.19	–0.98	–2.27	0.57

a *E*_f_, *E*_f0_ – *E*_f_, *E*_pd_, and *E*_pd0_ – *E*_pd_ stand for the Fermi energy, the shift in the Fermi energy with the adsorption of the corresponding adsorbate, the d or p band center, and the shift in the band center after the adsorption, respectively. All values are expressed in eV.

Remarkably, this information is useful to predict the magnitude of the effect of strains of a specific adsorbate on a specific surface without the need for any calculation with deformation. When the adsorbate heavily shifts the bands of the surface at zero strain, the effect of elastic strains in that adsorption process is going to be significant. In contrast, if the presence of the adsorbate on the unstrained surface does not notably modify the bands, the application of strains is not going to provoke big changes in the adsorption energy.

Similarly, for Pt(111), the Bader charge transfer was calculated for PtO_2_ in order to understand the variations of *E*_elec_, the nature of the interaction, and the balance of charges between the adsorbate and the surface. The charge transfers of each of the seven adsorbates into a surface of PtO_2_(110) are shown in Table 7. Negative values of charge transfer report a loss of electronic density, while positive values report a gain. The six atoms that conform the surface were labeled as Pt1, Pt2, O1, O2, O3, and O4. In this case, the adsorbates show two different adsorption sites: ONTOP_Pt_2 and ONTOP_O_1, which have been labeled as Ads and Ads′, respectively. In contrast with the case of Pt(111), where the surface donated charge for all the adsorbates, two different tendencies were found. H and N donate charge to the surface O (very electronegative) when the adsorption occurs on top of an oxygen atom (ONTOP_O_1, Ads′). On the contrary, the Pt surface atoms donate charge to the adsorbate if the adsorption occurs on top of a metal atom (ONTOP_Pt_2, Ads). The H atom donates the largest amount of charge to the surface, while the O atom receives the largest from it. This is in accordance with the electronegativity of the adsorbates. For the diatomic adsorbates (OH, CO, and NO), the O retains most of the charge donated from the surface, while H, C, and N lose charge.

**Table 7:**
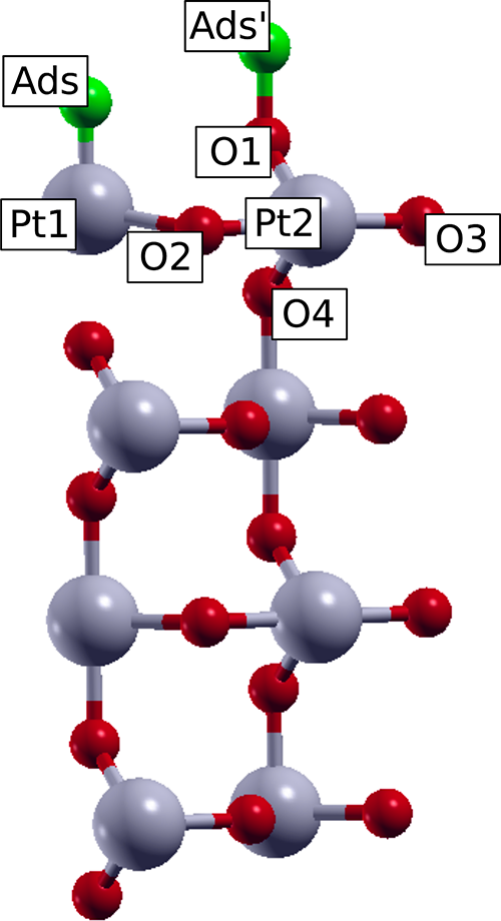
Charge Transfer for the 7 Adsorbates in a PtO_2_(110) Surface at ONTOP_Pt_2 (Ads) and ONTOP_O_1 (Ads′) Positions at Zero Strain^a^

system	Pt1 (*e*)	Pt2 (*e*)	O1 (*e*)	O2 (*e*)	O3 (*e*)	O4 (*e*)	adsorbate (*e*)	
H/PtO_2_(110)	–3.09	–3.09	1.20	2.54	2.23	0.92	–0.72 (ads′)	
N/PtO_2_(110)	–3.07	–3.07	0.89	2.56	2.50	0.71	–0.49 (ads′)	
O/PtO_2_(110)	–1.87	–1.98	0.71	0.87	0.87	0.92	0.54 (ads)	
C/PtO_2_(110)	–1.67	–1.84	0.79	0.88	0.88	0.93	0.12 (ads)	
OH/PtO_2_(110)	2.12	–1.96	0.74	–3.12	–3.12	0.92	1.45 (O)	0.45 (ads)
							–0.99 (H)	
CO/PtO_2_(110)	–1.72	–1.85	0.82	0.88	0.88	0.94	–1.72 (C)	0.05 (ads)
							1.77 (O)	
NO/PtO_2_(110)	2.16	–1.85	0.83	–3.12	–3.12	0.94	–0.27 (N)	0.16 (ads)
							0.43 (O)	

a The Pt1, Pt2, O1, O2, and O3 positions are shown in the scheme at the side of the table. All values are expressed in elemental units where *e* = −1. A positive value expresses a gain of negative charge, whereas a negative value expresses a loss of negative charge.

This analysis shows once again that the charge transfer between the surface and the adsorbate can be correlated with the variations of *E*_elec_ with strain, and the variation of *E*_elec_ with the applied strain increases with the charge donated from the surface to the adsorbate. For instance, the charge transfers from N and NO to the surface are −0.49 and 0.16*e*, respectively, and the effect of strain on *E*_elec_ is much higher in the case of N. In the cases of C/CO and O/OH, the charge donated from the surface to the adsorbate is similar, and the variations of *E*_elec_ with the applied strain are also comparable (Figure 6a). On average, the amount of charge transferred in PtO_2_ is lower than in Pt, as is the *E*_elec_, but the differences are small. Regarding the effect of strain in PtO_2_, the outcome is analogous to that for Pt: when subjected to tensile strains, the surface atoms are pushed further apart, thereby expanding the area of the adsorption site. This causes a decrease in the distance between the surface and the adsorbate, resulting in a higher level of charge transfer. Conversely, compressive strains have the opposite effect, increasing the distance between the adsorbate and the surface and reducing the extent of charge transferred. In conclusion, the bigger the amount of charge involved in the adsorption process, the higher the variation of energy the strain is going to provoke.

### 4.3 *E*_mech_ in Pt and PtO_2_

The mechanical contributions to the adsorption energy, *E*_mech_, due to the distortion of the lattice in the presence of the adsorbate are depicted in Figures 4b and 6b for Pt and PtO_2_, respectively. It should be noted that *E*_mech_ is much higher in the case of PtO_2_(110) surfaces (up to 0.7 eV) than in Pt(111) surfaces (up to 0.1 eV). In both cases, these contributions are practically independent of the applied elastic strain, indicating that they only depend on the distortion of the lattice induced by the presence of adsorbate. Even in the CO/PtO_2_, NO/PtO_2_, and OH/PtO_2_ cases, with changes in *E*_mech_ around 0.3 eV, a little bit higher than the commonly accepted 0.1–0.2 eV DFT errors, the variation in *E*_elec_ with strain is presumably more significant. The correlation between the surface–adsorbate distance and mechanism energy is not that clear in the case of PtO_2_ than in the case of pure Pt. Finding a metric in the former case is much more complicated, as when the adsorption happens in the PtO_2_ surface, all atoms—not only the ones close to the adsorption site—experience a variation in their position. However, even if not perfect, the proposed correlation can serve as an approximate approach to get a broad sense in how the mechanism energy would be.

**Figure 7:**
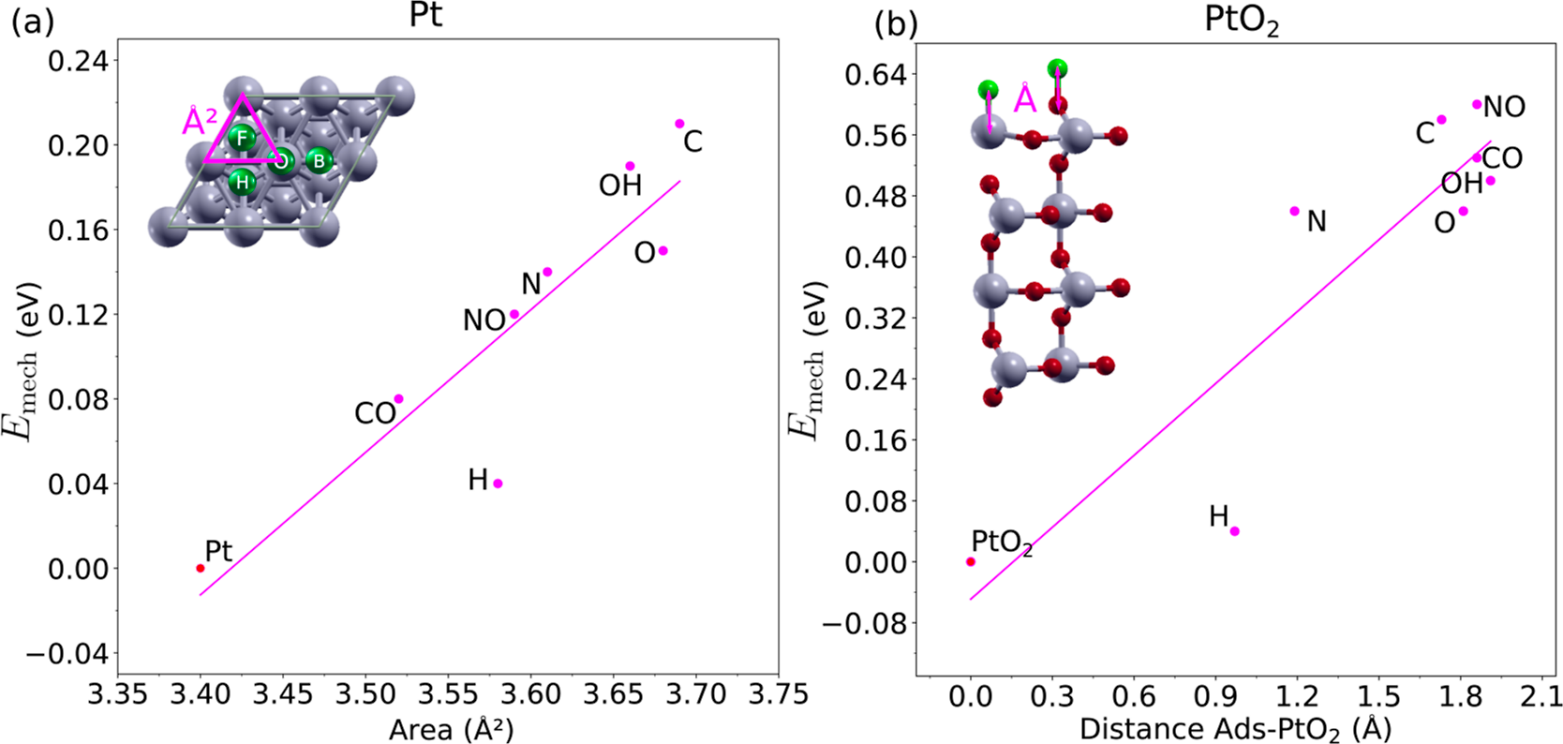
Relationship between the values of *E*_mech_ (eV) and the (a) area of the adsorption hole (Å^2^) in Pt(111) and the (b) surface–adsorbate distance (Å) in PtO_2_(110) for the seven adsorbates.

The adsorption process in Pt occurs in the hole between three surface atoms. As shown in Figure 7a, the magnitude of *E*_mech_ increases with the dimensions of the hole, and the larger the distortion, the larger *E*_mech_. This metric cannot be applied, however, to PtO_2_, as the adsorption occurs on top of Pt and O atoms and not in a hole. In this case, the presence of the adsorbate on the surface provokes a vertical displacement of the surface layer toward the adsorbate. *E*_mech_ is plotted in Figure 7b as a function of the distance between the adsorbate and the surface atom below. In general, longer distances are associated with higher *E*_mech_, as the surface atoms have to rearrange more to balance the shift up of the atom below the adsorbate. The lowest adsorption distance appears for H, where *E*_mech_ is also small. For the rest of the adsorbates, the atomic displacements are similar as are their values of *E*_mech_.

Finally, it should be noted that *E*_mech_ is higher in PtO_2_, as compared with Pt. This difference can be explained by the nature of the bonding. In the case of Pt, the interactions within the slab are metallic, whereas they are ionic in PtO_2_. The electrons are moving freely around the nucleus in the electronic cloud in Pt while they are localized toward the oxygen atoms in PtO_2_ due to the different electronegativity of Pt and O. Thus, the adsorption of an atom causes a higher distortion in the electronic cloud in the latter case, leading to a rearrangement of the atoms to find a most stable position.

## 5 Conclusions

The influence of elastic strain on the adsorption process onto Pt and PtO_2_ of seven adsorbates (H, C, N, O, CO, NO, and OH) was calculated by DFT simulations. Moreover, the adsorption energy was split into mechanical and electronic contributions. It was found that the adsorption energies decrease with tensile strains and increase with compressive strains in Pt and PtO_2_ for all adsorbates. In addition, the mechanical contribution to the adsorption energy was independent of the elastic strain and, thus, the effect of elastic strains on the adsorption energy was due to changes in the electronic structure.

The sensitivity of the electronic contribution to the adsorption energy of a given system (either X/Pt or X/PtO_2_) could be explained by the d-band model. The adsorbates that lead to a maximum shift in the d-band center also show the largest variation in the electronic contribution to the adsorption energy with the elastic strain. They are C, N, O, and OH in Pt and N and NO in the case of PtO_2_. On the contrary, H adsorption always shows the lowest sensitivity to the application of elastic strains.

Finally, the mechanical contribution to the adsorption energy could be associated with the distortion of the lattice in the presence of the adsorbate. It was very small in the case of Pt (<0.2 eV) and slightly higher (up to 0.6 eV) in the case of PtO_2_. This difference was attributed to the ionic bonding with the PtO_2_ slab, which enhances the distortion.

## Supporting Information Available

Adsorption energies for each adsorbate and for the mono and diatomic adsorbates in Pt and PtO_2_ under strain, phonon calculations, DFT functional test, charge transfer with strain for X/Pt(111), and PDOS of the X/Pt(111) and X/PtO_2_(110) adsorption processes (PDF)
