# Supplementary material for: Applicability of the d-Band Model to Predict the Influence of Elastic Strains on the Adsorption Energy of Different Adsorbates onto Pt and PtO2 Surfaces
Source: ACS Omega. 2024 Jun 24;9(27):29884–95. doi: 10.1021/acsomega.4c03830 (PMC11238222; doi:10.1021/acsomega.4c03830)
Supplement: Supplementary file 1 — ao4c03830_si_001.pdf [file ao4c03830_si_001.pdf]

Applicability of the d-Band Model to Predict the Influence of Elastic Strains on the Adsorption Energy of Different Adsorbates onto Pt and PtO_2 Surfaces
Supporting Information

Carmen Martínez-Alonso[a,b,*], Javier LLorca[a,c]

[a]*IMDEA Materials Institute, C/Eric Kandel 2, 28906 - Getafe, Madrid, Spain.*
[b]*Department of Inorganic Chemistry, Complutense University of Madrid, 28040 Madrid, Spain.*
[c]*Department of Materials Science, Polytechnic University of Madrid, E. T. S. de Ingenieros de Caminos, 28040 Madrid, Spain.*

Contents:

1. **S1**. Adsorption energies for each adsorbate.
2. **S2**. Adsorption energies for the monoatomic adsorbates in Pt under strain.
3. **S3**. Adsorption energies for the diatomic adsorbates in Pt under strain.
4. **S4**. Adsorption energies for the monoatomic adsorbates in PtO_2 under strain.
5. **S5**. Adsorption energies for the diatomic adsorbates in PtO_2 under strain.
6. **S6**. Phonon calculations.
7. **S7**. DFT functionals test.
8. **S8**. Charge transfer with strain for X/Pt(111).
9. **S9**. PDOS of the X/Pt(111) adsorption process.
10. **S10**. PDOS of the X/PtO2(110) adsorption process.
11. **References**

[*]To whom correspondence should be addressed: carmen.martinez@imdea.org

Published in ACS Omega

S1. Adsorption energies for each adsorbate

The different equations for the calculation of the adsorption energy with each of the adsorbates are shown below:

$$E_{\mathrm{adsH}} = E_{\mathrm{slab+H}} - (E_{\mathrm{slab}} + \frac{1}{2}E_{\mathrm{H_2}}) \quad (1)$$

$$E_{\mathrm{adsO}} = E_{\mathrm{slab+O}} - (E_{\mathrm{slab}} + \frac{1}{2}E_{\mathrm{O_2}}) \quad (2)$$

$$E_{\mathrm{adsN}} = E_{\mathrm{slab+N}} - (E_{\mathrm{slab}} + \frac{1}{2}E_{\mathrm{N_2}}) \quad (3)$$

$$E_{\mathrm{adsC}} = E_{\mathrm{slab+C}} - (E_{\mathrm{slab}} + E_{\mathrm{CH_4}} - 2E_{\mathrm{H_2}}) \quad (4)$$

$$E_{\mathrm{adsOH}} = E_{\mathrm{slab+OH}} - (E_{\mathrm{slab}} + (E_{\mathrm{H_2O}} - \frac{1}{2}E_{\mathrm{H_2}})) \quad (5)$$

$$E_{\mathrm{adsNO}} = E_{\mathrm{slab+NO}} - (E_{\mathrm{slab}} + E_{\mathrm{NO}}) \quad (6)$$

$$E_{\mathrm{adsCO}} = E_{\mathrm{slab+CO}} - (E_{\mathrm{slab}} + E_{\mathrm{CO}}) \quad (7)$$

S2. Adsorption energies for the monoatomic adsorbates in Pt under strain.

Table S 1: Adsorption energies for the monoatomic adsorbates in Pt under strain. All values are shown in eV.

System	Biaxial Strain (%)	E_{ads} (eV)
H/Pt	-5	-0.30
	-2	-0.39
	0	-0.49
	2	-0.56
	5	-0.60
O/Pt	-5	-1.63
	-2	-1.94
	0	-2.16
	2	-2.33
	5	-2.49
N/Pt	-5	0.73
	-3	0.55
	-1	0.37
	0	0.27
	1	0.18
	3	0.00
	5	-0.13
C/Pt	-5	2.59
	-3	2.41
	-1	2.15
	0	2.01
	1	1.88
	3	1.66
	5	1.51

S3. Adsorption energies for the diatomic adsorbates in Pt under strain.

Table S 2: Adsorption energies for the diatomic adsorbates in Pt under strain. All values are shown in eV.

System	Biaxial Strain (%)	E_{ads} (eV)
OH/Pt	-2	1.44
	0	1.19
	2	0.92
	5	0.62
NO/Pt	-5	-1.76
	-3	-1.83
	-1	-1.97
	0	-2.04
	1	-2.10
	3	-2.16
	5	-2.19
CO/Pt	-5	-1.51
	-3	-1.56
	-1	-1.68
	0	-1.74
	1	-1.79
	3	-1.84
	5	-1.87

S4. Adsorption energies for the monoatomic adsorbates in PtO_2 under strain.

Table S 3: Adsorption energies for the monoatomic adsorbates in PtO_2 under strain. All values are shown in eV.

System	Biaxial Strain (%)	E_{ads} (eV)
	-5	-0.70
	-3	-0.83
H/PtO_2	0	-1.01
	3	-1.18
	5	-1.30
	-5	-0.21
	-3	-0.30
O/PtO_2	0	-0.42
	3	-0.52
	5	-0.58
	-3	2.74
N/PtO_2	0	2.53
	3	2.29
	5	2.10
	-5	5.13
	-3	5.03
C/PtO_2	0	4.88
	3	4.72
	5	4.64

S5. Adsorption energies for the diatomic adsorbates in PtO_2 under strain.

Table S 4: Adsorption energies for the diatomic adsorbates in PtO_2 under strain. All values are shown in eV.

System	Biaxial Strain (%)	E_{ads} (eV)
	-5	2.02
	-3	1.89
OH/PtO_2	0	1.74
	3	1.62
	5	1.54
	-5	-0.16
	-3	-0.38
NO/PtO_2	0	-0.70
	3	-1.03
	5	-1.24
	-5	-0.94
	-3	-1.15
CO/PtO_2	0	-1.42
	3	-1.65
	5	-1.77

S6. Phonon calculations.

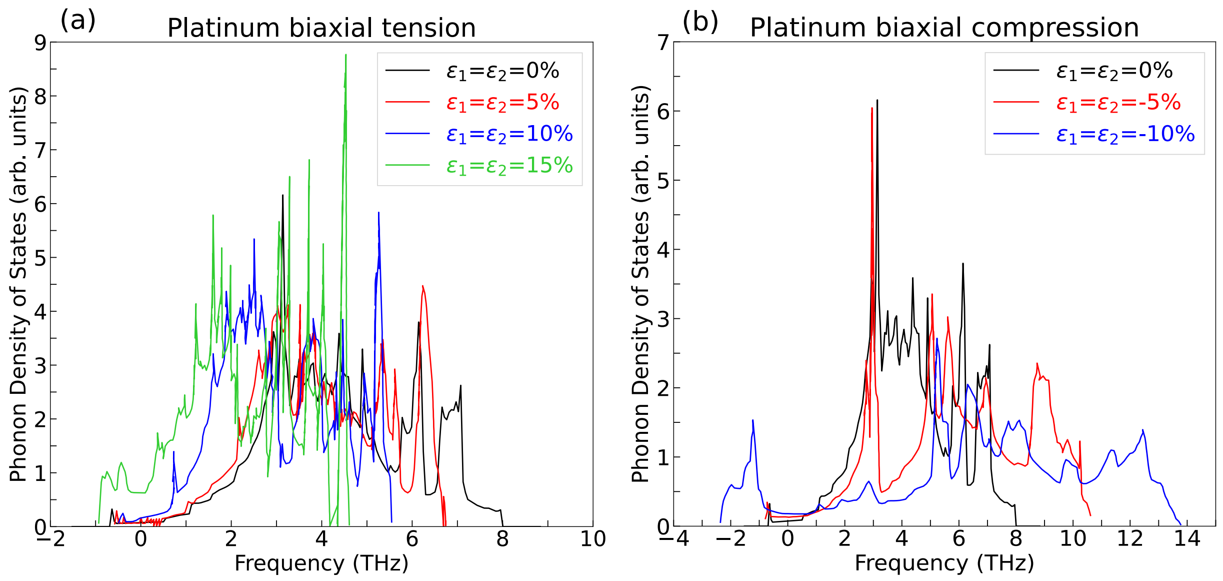

Figure S1: Phonon density of states for the (111) fcc Pt slab subjected to (a) biaxial tensile strains, and (b) biaxial compressive strains.

S7. DFT functionals test.

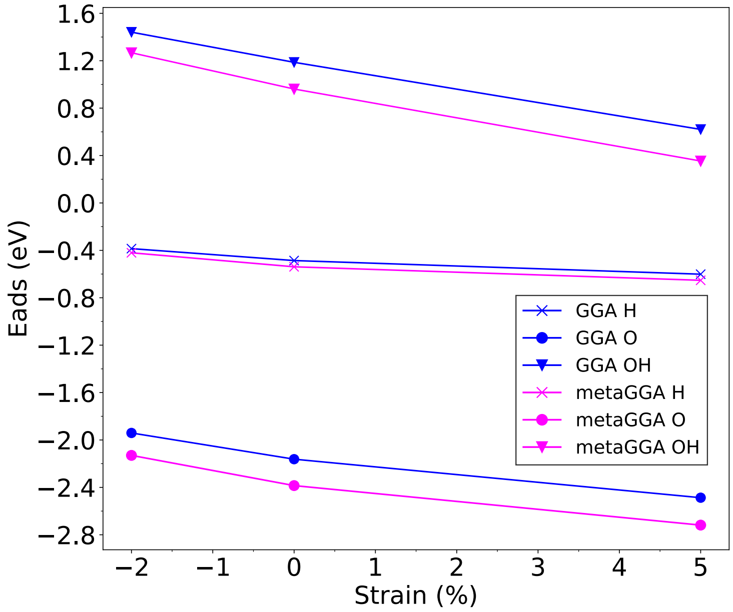

Figure S2: Adsorption energy of H, O, and OH in a Pt(111) slab as a function of biaxial strain. Blue curves represent the calculations performed with a GGA functional (PBE [1]) and magenta curves represent the calculations performed with a metaGGA functional (SCAN [2]).

S8. Charge transfer with strain for X/Pt(111).

Table S 5: Effect of strains in the charge transfer of the 7 different adsorbates onto a Pt(111) surface.

System	Strain (%)	Pt1 (e)	Pt2 (e)	Pt3 (e)	Pt4 (e)	Ads (e)	Ads-Pt distance (Å)
	-3	-0.02	0.05	0.02	-0.01	0.09	0.97
H/Pt(111)	0	-0.02	0.04	0.02	0.00	0.11	0.86
	5	0.01	0.07	0.03	0.01	0.12	0.68
	-3	-0.27	-0.29	-0.25	0.05	0.78	1.29
O/Pt(111)	0	-0.25	-0.28	-0.26	0.06	0.80	1.18
	5	-0.25	-0.26	-0.22	0.07	0.82	0.96
	-3	-0.18	-0.21	-0.19	0.06	0.49	1.14
C/Pt(111)	0	-0.17	-0.21	-0.19	0.06	0.50	0.96
	5	-0.15	-0.18	0.18	0.07	0.51	0.66
	-3	-0.26	-0.29	-0.28	0.06	0.77	1.20
N/Pt(111)	0	-0.26	-0.30	-0.28	0.06	0.80	1.07
	5	-0.26	-0.29	-0.28	0.08	0.84	0.76
	-3	-0.15	-0.17	-0.16	0.09	0.48	1.62
OH/Pt(111)	0	-0.14	-0.16	-0.16	0.08	0.47	1.46
	5	-0.13	-0.16	-0.14	0.09	0.48	1.31
	-3	-0.07	-0.11	-0.09	0.04	0.31	1.45
CO/Pt(111)	0	-0.07	-0.11	-0.08	0.05	0.29	1.37
	5	-0.06	-0.08	-0.07	0.06	0.33	1.26
	-3	-0.17	-0.19	-0.18	0.07	0.49	1.45
NO/Pt(111)	0	-0.16	-0.20	-0.17	0.07	0.50	1.37
	5	-0.14	-0.17	-0.17	0.09	0.53	1.26

S9. PDOS of the X/Pt(111) adsorption process.

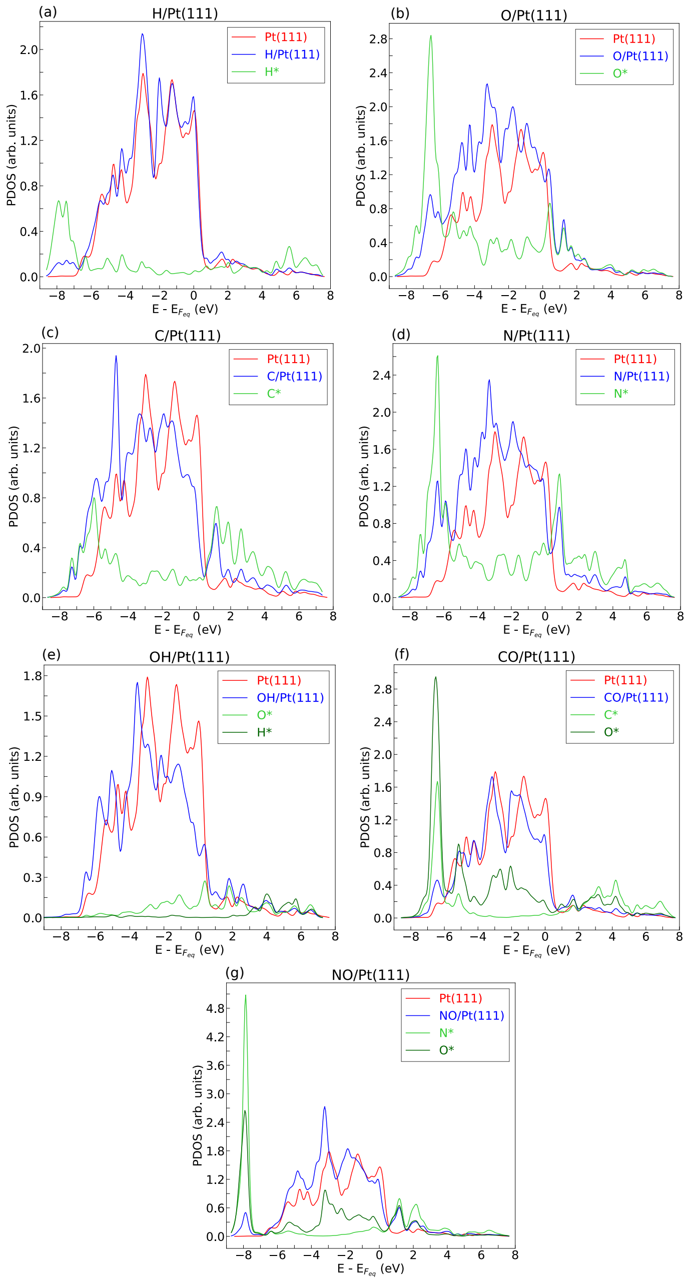

Figure S3: PDOS of (a) H, (b) O, (c) C, (d) N, (e) OH, (f) CO, and (g) NO adsorption into a Pt(111) surface. Red lines represent the PDOS of the platinum surface atoms of the clean slab, blue lines represent the PDOS of the platinum surface atoms with the adsorbate and green lines represent the PDOS of the adsorbed atoms.

S10. PDOS of the X/PtO_2(110) adsorption process.

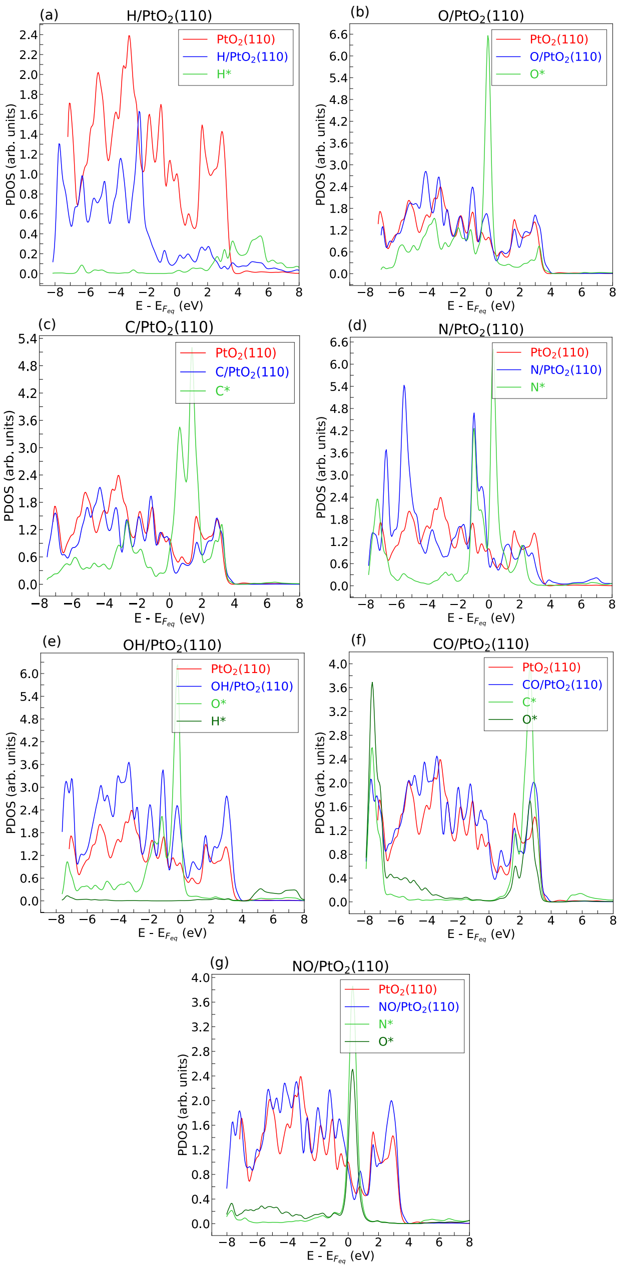

Figure S4: PDOS of (a) H, (b) O, (c) C, (d) N, (e) OH, (f) CO, and (g) NO adsorption into a PtO_2(110) surface. Red lines represent the PDOS of the PtO_2 surface atoms of the clean slab, blue lines represent the PDOS of the PtO_2 surface atoms with the adsorbate and green lines represent the PDOS of the adsorbed atoms.

References

[1] John P. Perdew, Kieron Burke, and Matthias Ernzerhof. Generalized gradient approximation made simple. *Phys. Rev. Lett.*, 77(18):3865–3868, October 1996.

[2] Jianwei Sun, Adrienn Ruzsinszky, and JohnP. Perdew. Strongly constrained and appropriately normed semilocal density functional. *Physical Review Letters*, 115(3), July 2015.
